# Haplotypes affecting stillbirth and fertility in Icelandic Dairy Cattle

**DOI:** 10.1007/s13353-025-00978-0

**Published:** 2025-06-02

**Authors:** Egill Gautason, Þórdís Þórarinsdóttir, Goutam Sahana

**Affiliations:** 1 Agricultural University of Iceland, Hvanneyri, Borgarbyggð, 311 Iceland; 2 The Icelandic Agricultural Advisory Center, Óseyri 2, Akureyri, 603 Iceland; 3 Center for Quantitative Genetics and Genomics, Aarhus University, C. F. Møllers Allé 3, Aarhus, 8000 Denmark

**Keywords:** Cattle, Stillbirth, Fertility, Homozygous haplotype deficiency, Recessive lethal

## Abstract

In the last decades, the rate of stillbirths in the Icelandic Dairy Cattle population has increased. Some of these stillbirths may be caused by recessive lethal mutations segregating in the population. These alleles can be identified by detecting homozygous haplotype deficiency (HHD) in genotyped animals. The aim of this study was to find genomic regions affecting stillbirth and fertility in the Icelandic Dairy Cattle population. We analysed genotypes from 20,557 animals with 35,481 autosomal markers. We identified HHD segments and estimated their effects on stillbirths and insemination failure, measured as non-return rates. We conducted genome-wide association studies (GWAS) for stillbirth and five fertility traits: interval from first to last inseminations, conception rate, number of inseminations, calving interval and infertility. While no GWAS association reached the genome-wide significance threshold, some of the top signals co-located with HHD haplotypes. A total of 19 haplotypes significantly either decreased fertility, or increased incidence of stillbirths, or both. Two HHD regions on BTA13: 43,577,221–59,026,521 and BTA8: 83,276,598–84,472,391 were associated with both lower fertility and higher incidence of stillbirths. We found no evidence of large structural variations in or around the HHD regions, suggesting that these signals are likely due to single loss-of-function mutation or small structural variations. Further research should focus on exploring these regions using whole genome sequence data.

**Supplementary Information:**

The online version contains supplementary material available at 10.1007/s13353-025-00978-0.

## Introduction

Icelandic Dairy Cattle is a dairy breed native to Iceland. Substantial genetic gains have been achieved for several breeding goal traits in the population through a well-executed breeding program that incorporates artificial insemination, best linear unbiased prediction (BLUP) for prediction of breeding values, and a total merit index for selection (Sigurdsson and Jonmundsson 2011). Although the breeding program has been effective in enhancing most traits contributing to the total merit index, some traits have changed unfavourably. In particular, the incidence of calf stillbirths has steadily increased over the recent decades, particularly among primiparous cows. In the 1990s, the calf mortality rate rose from 10 to 15% (Benjamínsson 2007). The underlying cause of the high stillbirth rate in Icelandic Cattle remains unclear despite substantial research (Jónsson, 2008; for an English summary, see Hardarson 2012).

Stillbirths in Icelandic Cattle may be partly attributed to loss-of-function genetic mutations that are lethal in homozygous state. Such recessive lethal mutations can be identified by detecting homozygous haplotype deficiency (HHD) (VanRaden et al. 2011a). Regions with HHD can also cause infertility if the foetus dies during gestation or soon after conception. As a follow-up, the effects of candidate recessive lethal haplotypes can be estimated on genotyped animals that have a phenotypic record for stillbirth and fertility.

Fertility has been part of the total merit index of Icelandic Dairy Cattle since 1993. Despite that, fertility has shown a declining trend (Þórarinsdóttir et al. 2021). Initially, fertility was evaluated based on calving interval (CI), but in 2021, a new model was implemented that included conception rate (CR), interval from first to last insemination (IFL) and interval from calving to first insemination (Þórarinsdóttir et al. 2021). A particularly alarming trend has been the rising incidence of heifer infertility, when heifers are culled without ever conceiving.

Intensive selection in the Icelandic Dairy Cattle population has caused inbreeding rates similar to those observed in other dairy cattle populations (Gautason et al. 2021a). Under the current breeding plan, it is most likely that high inbreeding rates will continue in the Icelandic Cattle population in the coming years, resulting in higher number of calves homozygous for recessive lethal mutations. Therefore, it is crucial to identify deleterious recessive alleles segregating in the population, to avoid their dissemination in the population, and to allocate matings to avoid homozygous calves. Since large-scale genotyping was introduced for routine genomic selection in cattle, haplotypes causing recessive disorders have been identified in several breeds (Cai et al. 2023; Fritz et al. 2013; Jenko et al. 2019; Pausch et al. 2015; Sahana et al. 2013; VanRaden et al. 2011a).

The high stillbirth rate in Icelandic Cattle is both economically damaging and a serious animal welfare concern, while fertility is an important economic trait. Therefore, the aim of this study was to identify genetic variants affecting stillbirth and fertility in Icelandic Cattle.

## Methods

### Genotypes

Genotype data was acquired from the database of the Farmer’s Association of Iceland. The animals were genotyped with Illumina BovineSNP50 version 2 and 3 or BovineHD SNP chip (Illumina, San Diego, USA) (Gautason et al. 2021b), and the EuroG MD chip (Boichard et al. 2018). The markers were mapped to the ARS-UCD1.2 cattle genome assembly (Rosen et al. 2020) and only autosomal markers were used. We removed SNP markers that were missing in more than 5% of animals and removed animals with call rate lower than 95%. Only animals with registered pure Icelandic Cattle ancestry were used. This resulted in 20,557 animals with 43,143 common markers, 870 of which were monomorphic. For GWAS, we removed animals with heterozygosity 3 standard deviations above the mean, markers deviating from Hardy–Weinberg proportions (P < 1e-6), and markers with MAF lower than 0.01. After these filtering steps, 35,481 markers remained for GWAS.

### Phenotypes

Phenotype data was obtained from the database of the Farmer’s Association of Iceland. The data included the pedigree of Icelandic Cattle, calving registrations that were recorded by farmers between January 2004 and August 2023, insemination data registered by AI technicians from 2008 until September 2024, and registered culling reasons. The insemination records and stillbirth records were separately filtered in line with the procedures used in the routine genetic evaluation as described below. Because the data are from separate sources and the traits in question different, the filtering procedures were not exactly the same. The extracted pedigree contained 677,129 animals. We added all stillborn calves to the pedigree and then traced it to animals with records and pruned the pedigree three generations back using DmuTrace Version 2 (Madsen 2010). After this filtering, the pedigree contained 472,743 individuals.

#### Stillbirths

The birth registrations included the fate of the calf in several categories. The following categories were defined as stillbirths: *stillborn*, *died during calving*, and *died within 24 h of calving*. Abortions were not considered as stillbirths. Each calving was assigned 0 for a dead calf, and 1 for a live calf. We removed twin calvings and used up to four calvings for each dam for GWAS, and up to five calvings for analysis of haplotype effects. We only used calving records for which the cow was more than 19 and less than 37 months old at first calving. These limits were set to remove extreme cases, as cows younger than 20 months and older than 36 months have substantially increased frequency of stillbirths.

#### Fertility

Before filtering, the dataset contained information on 168,940 animals and 697,309 artificial inseminations performed between 2008 and 2024. Only inseminations of maiden heifers (lactation 0) and the first three lactations (1–3) were included. We used these records to derive four fertility traits, namely conception rate (CR), calving interval (CI), interval from first to last insemination (IFL), and number of inseminations per service period (AIS). Our methods followed those described by Þórarinsdóttir et al. (2021), which are also used in the routine genetic evaluation. Conception rate is a measure of whether a cow conceived in her first insemination or not. CR was defined as a binary trait and computed based on insemination and calving records. CR was defined in a way that if IFL was in the interval 0–4 days and the animal calved 260–302 days later, then CR was set to 1 (success). If IFL was 5 days or more and the animal calved 260–302 days later then CR was set to 0 (failure). CR was also set to 0 if the cow was inseminated at least once but did not calve. Before analysis, the data was filtered to exclude animals with errors in recording of inseminations or calvings. While editing data, intervals were kept if within the following limits: age at first calving (550–1100 days), CI (280–600 days), age at first insemination (270–900 days), interval from calving to first insemination (20–230 days), IFL (0–365 days), interval from calving to last insemination (20–365 days), gestation length (260–302 days), and AIS (1–8). Records for lactations 2 and 3 were excluded if information about lactation 1 was unavailable and records for lactation 3 were excluded if information on lactation 1 or 2 was missing.

We also used insemination records to calculate non-return-rates at 56 days (NRR56). We excluded insemination records if the cow was re-inseminated less than 3 days after previous insemination. Records were also removed if gestation length, that is the interval from first insemination to calving, was shorter than 266 days. A cow was assigned a value of 1 if she was not re-inseminated within 56 days of her first insemination. Otherwise, if the cow was inseminated in the interval from day 3 to day 55, the cow was assigned 0. We further filtered the data to ensure that each fixed effect class contained at least 10 observations for all fertility trait analysed.

### Homozygous haplotype deficiency

We used the software findhap version 3 (VanRaden et al. 2011b; 2015) to construct haplotypes from the genotype data. We constructed haplotypes of 75 and 25 markers length. The program was run in three steps, first dividing the genotype data into 600 marker segments, then 212 markers and finally identifying haplotypes with length up to 75 or 25 markers. Haplotypes with frequency lower than 1% for the 75-marker haplotypes and 5% for the 25-marker haplotypes were discarded from further analysis. For each haplotype, we counted the number of homozygous individuals observed in the genotype data and used haplotypes with zero observed homozygous individuals for further analysis. Following VanRaden (2011a), we used two methods to estimate the number of expected homozygous animals, the simple method based on carrier frequency assuming random mating and another approach based on the carrier status of mating pairs. For the simple method, we identified the number of expected homozygous individuals for each haplotype as the squared carrier frequency divided by 4, multiplied with the number of genotyped animals. For the mating method, we used the pedigree to estimate the probabilities of observing homozygous individuals. For each genotyped animal, we checked whether the sire and the dam were genotyped. If both were genotyped, we counted the instances when both sire and dam were carriers. If the dam was not genotyped, we checked whether the maternal grand sire was genotyped. We counted the number of instances when both sire and maternal grand sire were carriers. Then the expected number of homozygous animals was computed as the sum of (carrier sire) × (carrier dam), and (carrier sire) × (carrier maternal grand sire) matings divided by 4. This method assumes that the carrier frequency in maternal grand sires and maternal grand dams is equal. For the simple method, probabilities of observing 0 homozygotes were computed as $P={\left(1-{C}^{2}/4\right)}^{N}$ where C was carrier frequency and N was number of genotyped animals. For the mating method, the probability was computed as 0.75 raised to the power of the sum of (carrier sire) × (carrier dam) and (carrier sire) × (carrier maternal grand sire) matings (VanRaden et al. 2011a). The haplotypes received an ID based on their position and haplotype number within position as defined by the software findhap.

### Haplotype effects

#### Stillbirth

We used the following statistical model to estimate the effect of HHD haplotypes on stillbirth. We only used calving records where both sire and dam, or both sire and maternal grand sire were genotyped. This resulted in 215,989 calving records. We applied logistic regression using the following model in R (R Core Team 2019) version 4.4.0:

1 $$\begin{array}{c}{Y}_{ijkl}=\mu +{Parity}_{i}+{Sex}_{j}+{Year\times Month}_{k}+\beta \times \left({P}_{hom-fetus}\right)+{e}_{ijkl}\end{array}$$

where $Y$ was stillbirth, $\mu$ was the intercept, *Parity* was parity of the dam taking values from 0 (for maiden heifers) to 4, *sex* was the sex of the calf, and $Year\times Month$ was year and month of calving. ${P}_{hom-fetus}$ was computed with the following equation if the dam and sire were genotyped:

2 $$\begin{array}{c}{P}_{hom-fetus}=0.5{\times P}_{carrier\, sire}\times 0.5\times {P}_{carrier\, dam}\end{array}$$

If the sire and maternal grand sire were genotyped, the following equation was used:

3 $$\begin{array}{c}{P}_{hom-fetus}=0.5{\times P}_{carrier\, sire}\times 0.25\times {P}_{carrier\, maternal\, grand\, sire}\end{array}$$

${P}_{carrier\, sire}$, ${P}_{carrier\, dam}$ and ${P}_{carrier\, maternal\, grand\, sire}$ took values of 0 or 1 depending on their carrier status.

### Rate of insemination failure as a function of carrier status

We did an analysis similar to Kadri et al. (2014) to test the effect of mating type on insemination failure measured as NRR56. The final dataset contained 136,475 insemination records with values for NRR56. We defined four classes of mating depending on carrier (C) and non-carrier (NC) status: (1) NC sire × NC maternal grand sire, (2) NC sire × C maternal grand sire, (3) C sire × NC maternal grand sire, (4) C sire × C maternal grand sire. The probability of both parents to be carriers for the putative lethal allele is (1) 0, (2), 0, (3) p, and (4) $\frac{0.5}{(1-0.5p)}$, where p is the frequency of the haplotype. The probability of the conceptus to be homozygous is the probability of both parents being carriers multiplied with 0.25: (1) 0, (2), 0, (3) 0.25p, and (4) 0.25$\frac{0.5}{(1-0.5p)}$. Assuming that the allele is lethal in homozygous state, we expect that reproductive failure is increased by the probability (1) 0, (2), 0, (3) 0.25*p*(1-*f*), and (4) 0.25 $\frac{0.5}{\left(1-0.5p\right)}\left(1-f\right)$ (Kadri et al. 2014). The background reproductive failure, *f*, was computed as the weighted mean of the average NRR56 value for mating types 1 and 2. We used the following linear mixed model to estimate the effects of haplotypes on insemination failure:

4 $$\begin{array}{c}{Y}_{ijkl}=\mu +{Parity}_{i}+{Year}_{j}+{Mating\, type}_{k}+{u}_{l}+{e}_{ijkl}\end{array}$$

where *μ* was the mean, *Parity* was the fixed effect of parity 0, 1, 2, or 3, and *Year* was the fixed effect of year of insemination. *Mating type* was the fixed effect of mating type and took values of 1, 2, 3 or 4 depending on the genotype of the sire and genotype of the sire of the dam. *u* was the random additive genetic effect of the inseminated cow, with distribution ~ N(0, **A**${\upsigma }_{u}^{2}$) where **A** was the numerator relationship matrix and *e* was a random residual effect with distribution ~ N(0, **I**${\upsigma }_{e}^{2}$). We estimated variance components and fit the model using Average information restricted maximum likelihood (AIREML) (Jensen et al. 1997) using the function dmuai of the DMU software package version 6, release 5.6 (Madsen & Jensen 2013). We plotted results using ggplot2 (Wickham 2016) version 3.5.1.

### Phenotypes for genome-wide association studies

We used linear mixed models to construct corrected phenotypes for genotyped cows with observations for stillbirth and fertility. We estimated variance components and predicted breeding values (EBV) using AI-REML in DMU.

### Stillbirth

To estimate variance components for stillbirth, we applied more strict filtering procedures in addition to those listed in the Phenotype section. We used calving records from 2009 to 2023. We removed all records within fixed year classes with 20 or fewer observations. We only included herds that were present throughout the period to avoid any bias due to management changes for herds starting or leaving production. We constructed a herd-year variable and removed herds for which there were fewer than 4 calvings in any herd-year class. This resulted in 223,179 records for estimation of variance components and 309,345 records in the dataset used to predict breeding values. On average, 26.0% of heifer calvings, and 9.2% of later calvings in the data resulted in stillborn calves. We defined 21 unknown parent groups to model the genetic trend in unknown sires and dams. These groups were fit as random effects in the models. The following linear bivariate model was applied:

$${y}_{ihdmskl}^{1}={H5Y}_{h}+{D}_{d}+{M}_{m}+{S}_{s}+{HY}_{k}+{m}_{l}+{u}_{i}+{e}_{ihdmskl}$$

5 $$\begin{array}{c}{y}_{ihdmsklo}^{2}={H5Y}_{h}+{D}_{d}+{M}_{m}+{S}_{s}+{HY}_{k}+{m}_{l}+{u}_{i}+{PE}_{lo}+{e}_{ihdmsklo}\end{array}$$

where *y* is the phenotype of calf *i*, *H5Y* is the fixed effect of herd by calving year group, where the groups were defined by five year periods, *D* is fixed effect of age of dam at calving in months, *M* is fixed effect of month of calving, *S* is fixed effect of the sex of calf, *HY* is the random effect of herd by year of calving, *m* is the random additive genetic maternal effect of dam, and *u* is the random additive genetic effect of the calf, *PE* is the random effect of dam’s permanent environment, and *e* is the random residual effect. Effects of *m* and *u* were assumed to be drawn from.

 ~ N $$\left(0, \mathbf{A}\otimes \left[\begin{array}{cc}\begin{array}{cc}{\upsigma }_{m1}^{2}& {\sigma }_{m1u1}\\ {\sigma }_{m1u1}& {\upsigma }_{u1}^{2}\end{array}& \begin{array}{cc}{\sigma }_{m1m2}& {\sigma }_{m1u2}\\ {\sigma }_{u1m2}& {\sigma }_{u1u2}\end{array}\\ \begin{array}{cc}{\sigma }_{m1m2}& {\sigma }_{u1m2}\\ {\sigma }_{m1u2}& {\sigma }_{u1u2}\end{array}& \begin{array}{cc}{\upsigma }_{m2}^{2}& {\sigma }_{m2u2}\\ {\sigma }_{m2u2}& {\upsigma }_{u2}^{2}\end{array}\end{array}\right]\right)$$ where ${\upsigma }_{u}^{2}$ and ${\upsigma }_{m}^{2}$ are the additive genetic variances for individual and maternal effects, and **A** is the numerator relationship matrix. The random effect of *HY* had distribution ~ N $$\left(0, \mathbf{I}\otimes \left[\begin{array}{cc}{\upsigma }_{HY1}^{2}& {\upsigma }_{HY12}\\ {\upsigma }_{HY12}& {\upsigma }_{HY2}^{2}\end{array}\right]\right)$$ where **I** is an identity matrix, the permanent environmental effect was assumed to be drawn from ~ N(0, **I**${\upsigma }_{PE}^{2}$), and the residual effect was assumed to be drawn from ~ N $$\left(0, \mathbf{I}\otimes \left[\begin{array}{cc}{\upsigma }_{e1}^{2}& 0\\ 0& {\upsigma }_{e2}^{2}\end{array}\right]\right)$$. The superscript denotes whether the dam gave birth to her first calf (1) or subsequent calvings (2). The phenotype y was set to 0 for stillbirths and 1 for live calves. The solutions for *m* and *u* gave predicted breeding values for four traits: daughter stillbirth at 1st calving (DSB1), sire stillbirth at 1st calving (SSB1), daughter stillbirth at calving 2–4 (DSB2) and sire stillbirth at calving 2–4 (SSB2). We computed the phenotype of animal *i* for stillbirth corrected for environmental effects as the sum of the predicted breeding value, permanent environment (for DSB2), and residual:

6 $$\begin{array}{c}{y}_{DSB1,i}^{*}={EBV}_{DSB1,i}+{e}_{SB1}\\ {y}_{DSB2,i}^{*}={EBV}_{DSB2,i}+{PE}_{DSB2,i}+{\overline{e} }_{SB2}\\ {y}_{SSB1,i}^{*}={EBV}_{SSB1,i}+{e}_{SB1}\\ {y}_{SSB2,i}^{*}={EBV}_{SSB2,i}+{e}_{SB2}\end{array}$$

where EBV was the predicted breeding value and ${e}_{SB1}$ and ${e}_{SB2}$ were the residuals according to model 5. For ${e}_{SB2}$, we used the mean of the residuals $\left({\overline{e}}_{SB2}\right)$ when the cow had more than one calving. This resulted in a total of 9548 and 9313 animals with corrected phenotypes for direct stillbirth and maternal stillbirth, respectively. We computed the heritability as ${h}_{DSB1}^{2}=\frac{{\sigma }_{m1}^{2}}{{\sigma }_{m1}^{2}+{\sigma }_{u1}^{2}+{\sigma }_{e1}^{2}+2{\sigma }_{m1u1}}$ and ${h}_{SSB1}^{2}=\frac{{\sigma }_{u1}^{2}}{{\sigma }_{m1}^{2}+{\sigma }_{u1}^{2}+{\sigma }_{e1}^{2}+{2\sigma }_{m1u1}}$ and similarly for DSB2 and SSB2.

### Fertility

The following linear repeatability mixed model was used for AIS and IFL:

7 $$\begin{array}{c}y=HY+Age+IYM+Parity+u+PE+e\end{array}$$

where *HY* was the fixed effect of herd by year. Year of birth was used for maiden heifers and calving year was used for older cows, *Age* was fixed effect of age of cow in months. Age at first insemination was used for heifers and age of calving was used for older cows. *IYM* was the random effect of insemination year-by-month, *u* was the random additive genetic effect of the animal with distribution ~ N(0, **A**${\upsigma }_{u}^{2}$), *PE* was the permanent environmental effect of the cow, with distribution ~ N(0, **I**${\upsigma }_{PE}^{2}$), and *e* was a random residual effect with distribution ~ N(0, **I**${\upsigma }_{e}^{2}$). For CR, we used the model in Eq. (7) but also included the fixed effect of technician at insemination. For CI, we used the following model:

8 $$\begin{array}{c}y=HY+Age+Parity+u+PE+e\end{array}$$

where *HY* was herd by year of calving and *Age* was age at calving in months, and other terms were the same as above. We used the same pedigree and unknown parent groups as for stillbirth. We then computed the corrected phenotype for these traits as the sum of EBV, PE, and mean residual:

9 $$\begin{array}{c}{y}_{i}^{*}={EBV}_{i}+{PE}_{i}+{\overline{e} }_{i}\end{array}$$

where ${\overline{e} }_{i}$ was the mean residual for animal *i*. We used 6406, 6410, 6407, and 5591 animals for IFL, AIS, CR, and CI. We also constructed a phenotype for the trait infertility. We obtained records of cows that had never calved and were registered as having been culled due to infertility. These cows were assigned 0 and cows that had calved were assigned 1. There were 107 cows assigned 0 and 10,106 assigned 1. We did not predict breeding values for infertility but used the raw phenotype for GWAS.

### Genome-wide association analysis

The genome-wide association studies were conducted using the software LDAK version 5.2 (Speed et al. 2012). We used a linear mixed model regression for all traits:

10 $$\begin{array}{c}{y}_{i}^{*}=\mu +b{g}_{i}+{u}_{i}+e\end{array}$$

where ${y}_{i}^{*}$ was phenotype of animal *i* as described above, *b* was the allele substitution effect of the SNP, *g* is the number of copies of the allele in animal *i*, *u* is the polygenic effect of individual *i* with distribution ~ N(0, **G**${\upsigma }_{u}^{2}$) where ${\upsigma }_{u}^{2}$ is the polygenic genetic variance and **G** is the genomic relationship matrix, and *e* is the random residual for animal i, with distribution ~ N(0, **I**${\upsigma }_{e}^{2}$).

Genome-wide significance threshold was set to 1.41e-6 with a Bonferroni correction for 35,481 tests. We used the arguments –calc-kins-direct GRM –ignore-weights YES –power −1 with LDAK to set up the genomic relationship matrix using 35,481 markers and 20,328 animals. We used the arguments –linear and –grm to run the GWAS. We considered an association suggestive if it was among the 10 markers with the lowest *P*-values for each trait even though the association had not reached the significant threshold after Bonferroni multiple testing correction. We checked whether each of these suggestive markers was within 1 Mb of an HDD haplotype, and whether it was in linkage disequilibrium (LD) with that of HDD haplotype. We used PLINK (Purcell et al. 2007) to compute LD and assumed that *r*^2^ greater than 0.5 indicated linkage. For markers co-locating with HHD haplotypes, we identified the protein-coding genes closest to the marker according to the ARS-UCD 1.2 genome build on ensembl (Yates et al. 2020). We plotted the *P*-values by chromosomes and the expected and observed distribution of *P* values using the R package ggmanh (Lee & Lee 2024).

### Search for chromosomal deletion

To look for candidate deletion regions, we computed probability of deviation from Hardy–Weinberg proportions using the *–hardy* command in PLINK. For each HHD region, we plotted *P*-values for deviation from Hardy–Weinberg proportions, and the mean log R values of carriers and non-carriers, to look for indications of large deletions.

### Identification of HHD haplotypes

We present HHD haplotypes that fulfilled one or more of the following criteria:

1. Had a statistically significant effect on stillbirth according to Eq. (1).
2. Had significant effects on rate of insemination failure according to Eq. (4).
3. Co-located with an association according to GWAS as described above. A non-significant association was considered suggestive if markers were among the top 10 SNPs with lowest *P*-values.

## Results

A total of 65 haplotypes were identified as deficient in homozygosity (Online Resource 1). Nineteen haplotypes fulfilling one or more of the criteria set in this study are presented in Table 1. They are located on BTA1, BTA2, BTA3, BTA6, BTA8, BTA10, BTA13, BTA17, BTA18, BTA20, BTA26, BTA27 and BTA28.

**Table 1:** Segments of homozygous haplotype homozygosity that either have a significant effect on phenotype, or colocate with GWAS signals, or both

BTA	Haplotype ID			Random mating^c^		Carrier mating		Haplotype				
		GWAS^a^	Effects on phenotype^b^	Expected	P	Expected	P	frequency	Start position^d^	End position	Length (bp)	Length (SNP)
1	106–1		NRR56	78	7.2.E-35	146	8.1E-74	0.06178	148,694,674	150,065,342	1,370,668	25
2	136–1		Stillbirth	79	6.1.E-35	188	1.1E-94	0.06185	28,668,665	29,822,786	1,154,121	25
2	149–14		Stillbirth	64	1.2.E-28	104	1.9E-52	0.05587	48,993,143	50,938,882	1,945,739	25
2	51–13		Stillbirth	6	1.8.E-03	27	5.7E-14	0.01756	51,524,924	54,536,305	3,011,381	74
3	248–2		Stillbirth	54	2..E-24	74	7.8E-38	0.05139	66,616,695	67,750,849	1,134,154	25
3	258–1		Stillbirth	67	4.6.E-30	109	6.0E-55	0.05728	80,841,130	81,634,774	793,644	25
6	517–2		Stillbirth	93	4.7.E-41	193	4.7E-97	0.06713	99,313,329	100,180,758	867,429	25
8	667–1	CI	Stillbirth	59	1.7.E-26	133	3.4E-67	0.05368	83,276,598	84,472,391	1,195,793	25
10	806–1		Stillbirth	109	4.9.E-48	146	1.4E-73	0.0727	70,049,800	71,399,940	1,350,140	25
10	278–418	Infertility		12	4.8.E-06	7.5	1.8E-04	0.0244	95,001,362	99,358,600	4,357,238	75
13	994–1		Stillbirth, NRR56	66	1.5.E-29	90	1.9E-45	0.05677	47,952,032	49,634,265	1,682,233	25
13	336–37		NRR56	31	3.4.E-14	24	1.0E-12	0.03882	51,673,886	55,687,392	4,013,506	72
13	337–35		NRR56	18	1.5.E-08	19	3.2E-10	0.0296	55,717,888	59,026,521	3,308,633	72
17	1215–1		Stillbirth	150	2.7.E-66	225	3.6E-113	0.08554	23,265,033	25,619,746	2,354,713	24
18	1279–9		Stillbirth	69	6.0.E-31	120	1.4E-60	0.05813	39,658,283	40,819,008	1,160,725	25
20	1372–3		Stillbirth	113	4.6.E-50	180	2.0E-90	0.07423	34,329,841	35,540,903	1,211,062	25
26	1616–4		Stillbirth	75	1.8.E-33	191	2.6E-96	0.06051	17,953,795	19,260,069	1,306,274	25
27	1645–19		Stillbirth	70	5.7.E-31	183	4.68E-92	0.05816	9,598,079	11,736,207	2,138,128	25
28	567–176	DSB2, SSB2		7	8.9.E-04	10	1.01.E-05	0.01849	402,771	4,807,450	4,404,679	72

^a^ Indicates the traits for which GWAS results co-located with the homozygous haploptype deficient segment

^b^ Indicates whether the segment had statistically significant (*P* < 0.05) effects on stillbirth or non-return rates at 56 days

^c^ Expected number of homozygous individuals according to random and carrier mating and corresponding *P*-values

^d^ Position is according to mapping to the ARS-UCD 1.2 genome

### Haplotype effects on stillbirth and NRR56

#### Stillbirth

The following haplotypes significantly (*P* < 0.05) increased probability of stillbirth based on the logistic regression analysis following Eq. (1): hap136-1, hap149-14, hap51-13, hap248-2, hap258-1, hap517-2, hap667-1, hap806-1, hap994-1, hap1215-1, hap1279-9, hap1372-3, hap1616-4, and hap1645-19. Results for all haplotypes are in Online Resource 2.

### Rate of insemination failure

**Fig. 1:**
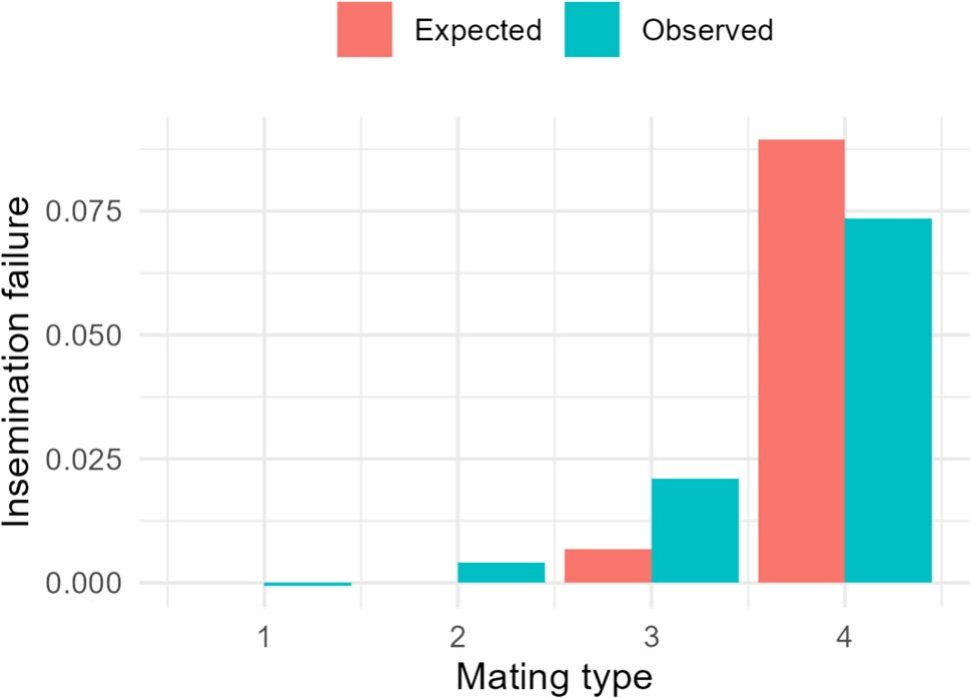
Observed and expected rate of extra insemination failure, measured as non-return rates at day 56, according to mating types: (1) non carrier (NC) sire × NC maternal grand sire, (2) NC sire × C maternal grand sire, (3) C sire × NC maternal grand sire, (4) C sire × C maternal grand sire

In total, four haplotypes had significant negative effects on NRR56: hap106-1, hap336-37, hap337-35 and hap994-1. Table 2 shows the BLUE estimates for different mating types and their corresponding z-scores and *P*-values. Three of them, hap336-37, hap337-35 and hap994-1, are located on BTA13 in the region 47,952,032–59,026,521. Hap336-37 had the largest effect. The BLUE estimates for hap336-7 for mating types 1, 2 and 3 were 0.074, 0.069 and 0.052 expressed as deviation from mating type 4. This means that 56 days after first insemination, when both sire and maternal grand sire are carriers, insemination failure is about 7% above mating type 1 and 2, and about 5% above mating type 3. The effect estimates for 337–35 were not significant for mating type 1 (*P* = 0.0588) but significant for mating type 2 (*P* = 0.002). Figure 1 shows the increased reproductive failure according to mating type, which was found by subtracting the BLUE value of mating type from the weighted average of the BLUE values of mating types 1 and 2 for hap336-37. The figure also shows the expected rate of insemination failure according to the background reproductive failure and frequency of the haplotype. The full table of haplotypes and corresponding BLUE values of mating types are presented in Online Resource 3.

**Table 2:** Haplotype effect on non-return rate at 56 days for mating types 1, 2, and 3 and their corresponding z scores and P-values. The effect estimates are expressed as deviations from mating type 4

BTA	Haplotype ID	Mating type^a^	Number of matings	Effect on NRR56	SE	z	P
1	106–1	1	85,295	0.014275	0.007215	1.978533	0.048
1	106–1	2	26,050	0.020733	0.006378	3.250821	0.0014
1	106–1	3	18,655	− 0.00154	0.007745	−0.1992	0.84
13	336–37	1	108,286	0.074106	0.013039	5.683424	< 0.0001
13	336–37	2	14,271	0.069397	0.012454	5.572408	< 0.0001
13	336–37	3	12,430	0.052498	0.013585	3.864529	0.00011
13	337–35	1	112,564	0.032435	0.017167	1.889335	0.059
13	337–35	2	12,393	0.050227	0.016213	3.097907	0.0019
13	337–35	3	10,671	0.01112	0.017666	0.629439	0.53
13	994–1	1	93,710	0.02416	0.00883	2.736236	0.0062
13	994–1	2	23,209	0.019835	0.00792	2.504271	0.012
13	994–1	3	15,679	0.009318	0.009417	0.989498	0.32

^a^Mating types were defined so: (1) non carrier (NC) sire × NC maternal grand sire, (2) NC sire × C maternal grand sire, (3) C sire × NC maternal grand sire, (4) C sire × C maternal grand sire

### Genome-wide association studies

No marker reached the genome-wide significance threshold. However, there were some suggestive associations. Table 3 shows the suggestive association signals co-locating with HHD haplotypes along with the protein coding genes closest to the associated marker. Table 4 presents variances and heritabilities of stillbirth and fertility traits. Test statistics for top ten markers for all traits are available in Online Resource 4. Figures showing plots of expected and observed distribution of *P*-values are available as Online Resource 5.

**Table 3:** Markers showing suggestive association with fertility related traits and co-locating with homozygous haplotype deficient regions. Closest protein-coding genes are according to ensembl (Yates et al. 2020)

BTA	Marker	Marker position	Haplotype	Haplotype position	Trait	Genes^a^
8	Hapmap48898-BTA-79943	84,123,194	667–1	83,276,598–84,472,391	CI	ECM2,CENPP
10	ARS-BFGL-NGS-23086	97,739,539	278–418	95,001,362–99358600	Infertility	FLRT2, ENSBTAG00000002900
28	BTB-01590721	618,487	567–176	402,771–4807450	DSB2	OR5 AS1, RHOU
28	BTB-01498660	640,998	567–176	402,771–4807450	SSB2,DSB2	OR5 AS1, RHOU

^a^ Closest protein-coding genes upstream and downstream of the associated markers according to ARS-UCD1.2 on ensembl

**Table 4:** Residual and additive genetic variances and heritabilities based on pedigree and the GWAS model for daughter stillbirth at 1 st calving (DSB1), sire stillbirth at 1 st calving (SSB1), daughter stillbirth at calving 2–4 (DSB2) sire stillbirth at calving 2–4 (SSB2), conception rate (CR), calving interval (CI), interval from first to last insemination (IFL), and number of inseminations per service period (AIS)

	Variances		Heritability	
	Additive genetic	Residual	Pedigree-based	Genomic
SSB1	0.015	0.162	0.081	0.046
DSB1	0.016	0.162	0.086	0.058
SSB2	0.0020	0.078	0.025	0.023
DSB2	0.0020	0.078	0.024	0.023
CR	0.0039	0.22	0.017	0.021
CI	92	2158	0.043	0.068
IFL	25	1774	0.014	0.022
AIS	0.020	1.0	0.020	0.023

#### Stillbirth

**Fig. 2:**
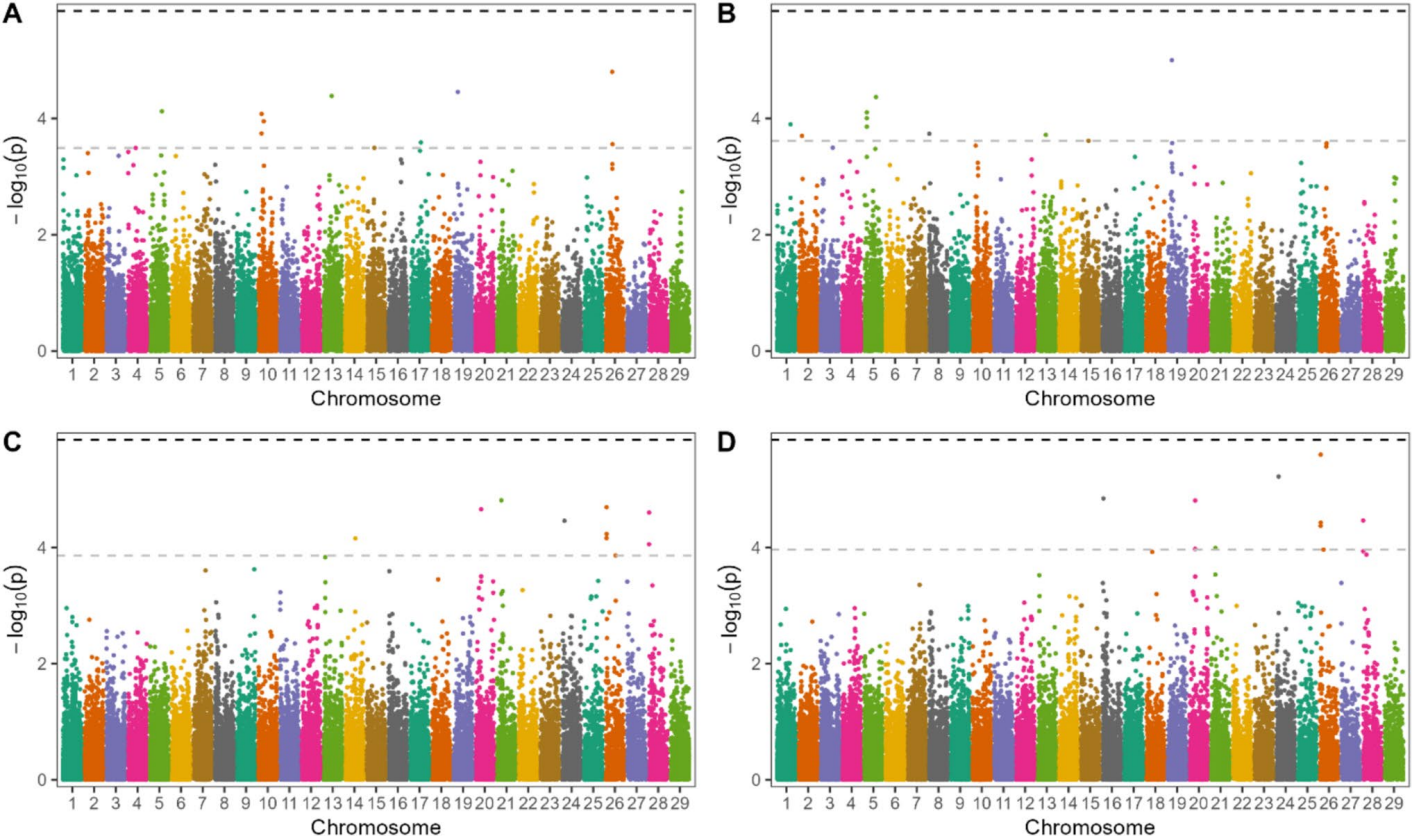
Manhattan plot for genome-wide association study for stillbirth. **A** daughter stillbirth at 1 st calving (DSB1), **B** sire stillbirth at 1 st calving (SSB1), **C** daughter stillbirth at calving 2–4 (DSB2) and (**D**) sire stillbirth at calving 2–4 (SSB2). The black dotted line denotes the Bonferroni-corrected significance threshold (*P* < 0.05), while the grey line indicates threshold for suggestive association, which was the 10 th lowest *P*-value observed

Figure 2 shows the results of association studies for stillbirth traits. Two suggestive signals on BTA28 co-located with hap567-176. A marker on BTA26: 19,823,316 was within 1 Mb of haplotype hap1616-4, but *r*^2^ values did not indicate linkage between the marker and haplotype. The genomic inflation factor was 1.023, 1.014, 1.000, and 1.014 for DSB1, SSB1, DSB2, and SSB2.

#### Fertility

**Fig. 3:**
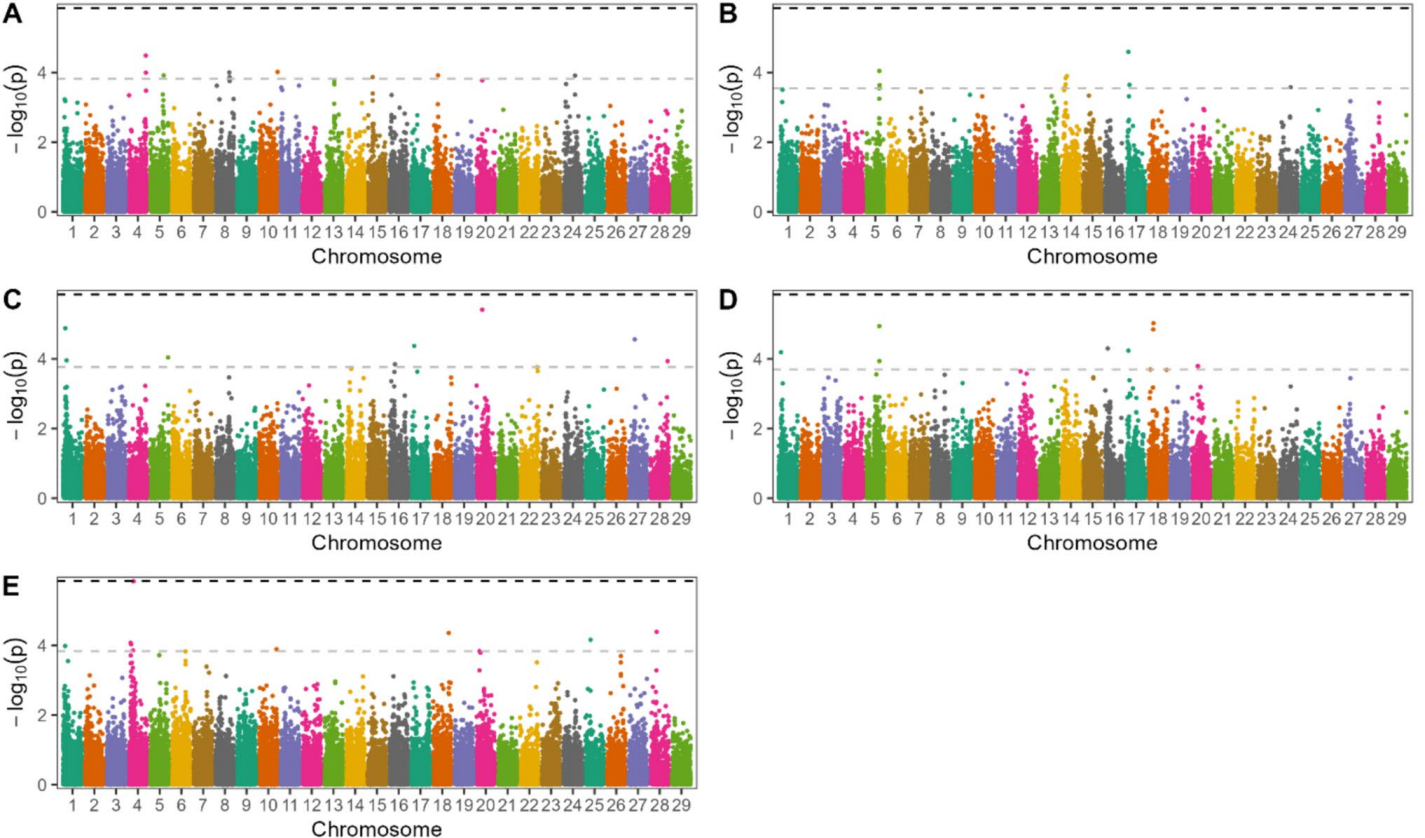
Manhattan plots for genome-wide association study for fertility traits: **A** calving interval (CI), **B** conception rate (CR), **C** interval from first to last insemination (IFL), **D** number of inseminations per service period (AIS), **E **Infertility. The black dotted line denotes the Bonferroni-corrected significance threshold (*P* < 0.05), while the grey line indicates threshold for suggestive association, which was the 10 th lowest *P*-value observed

The results of the association study for CI, CR, AIS, IFL and infertility are presented in Fig. 3. Two suggestive association signals co-located with HHD regions on BTA8 (CI) and BTA10 (Infertility). Hap667-1 on BTA8 co-located with a suggestive association with CI and significantly increased risk of stillbirth. Genomic heritability was 0.013 for infertility. The genomic inflation factor was 0.987, 1.01, 0.971, 0.993, and 0.997 for CI, CR, AIS, IFL, and infertility.

### Search for chromosomal deletion

We plotted deviations from Hardy–Weinberg proportions and log R intensities of markers in HHD regions but found no indication for presence of large deletions (results not shown).

## Discussion

A total of 65 haplotypes were identified as exhibiting homozygous haplotype deficiency. Seventeen of these haplotypes, located on 12 autosomes, significantly increased risk of stillbirth, or decreased NRR56, or both. Two haplotypes co-located with suggestive GWAS associations for stillbirth, and two haplotypes co-located with suggestive associations for fertility traits. The regions on BTA13 and BTA8 are especially interesting due to multiple evidence of recessive lethal mutations.

Four HHD haplotypes were identified in the region BTA13:43,577,221–59,026,521. Our analysis showed that for three of these haplotypes on BTA13, hap336-37, hap337-35 and hap994-1, carrier-by-carrier matings significantly increased insemination failure as measured by NRR56. For hap336-37, the observed increase in insemination failure was close to the theoretical expected values for recessive lethal mutations. These results indicate a mutation in this region on BTA13 that causes early embryonic lethality in Icelandic Dairy Cattle. Interestingly, carriers of hap994-1 also had significantly increased risk of stillbirths. It is possible that a recessive lethal allele in the region has incomplete penetrance, and that some of the homozygous foetuses survive until calving but tend to die soon before birth or during birth. Previous research in other breeds has found associations with female fertility traits in this region on BTA13 in Nordic Red, Nordic and US Holstein, and Jersey cattle (Höglund et al. 2014, 2015; Kiser et al. 2019; Sahana et al. 2010). Additionally, Shan et al. (2020, 2022) found a mutation in the ABHD16B gene (BTA13: 53,957,078–53,958,761) in Holstein Cattle that caused male infertility.

Hap667-1 on BTA8: 83,276,598–84,472,391 affected stillbirth and a marker on BTA8: 84,123,194 had a suggestive association with CI. A mutation in the IARS gene (BTA8: 83,837,389–83,918,620) is the cause of perinatal weak calf syndrome, which causes calf death and both fetal and embryonic lethality in Japanese Black Cattle (Hirano et al. 2013, 2016). A SNP marker on BTA8:84,474,299 has been associated with daughter pregnancy rate in US Holstein (Cole et al. 2011). Hap1616-4 on BTA26 both negatively affects stillbirth and co-located with a GWAS association for stillbirth.

A marker on BTA26:19,823,316 showed suggestive association with DSB1. However, the marker does not seem to be in LD with hap1616-4 on BTA26: 17,953,795–19,260,069. The marker, which was the top hit for DSB1, is close to the HPSE2 gene location (BTA26:19,540,093–19,813,443). Research in mice model has shown that homozygous knockout mutants for the gene had lower body weight and died within one month of birth (Guo et al. 2015). Therefore, a loss-of-function mutation in HPSE2 is a probable candidate cause of stillbirths in the Icelandic Cattle population, but LD suggests that there might be two independent factors affecting survival in the region. Kiser et al. (2019) found a locus associated with conception rate in US Holstein heifers on BTA26:19,200,857, adding further evidence that the region affects female fertility in cattle.

Two HHD haplotypes, hap977-1 on BTA13 and hap1244-1 on BTA17, have a frequency of 11.4% and 10.1%. They have an extremely large deficiency of homozygotes but did not have statistically significant effects on either stillbirth or fertility. However, hap977-1 had a numeric effect (*P* = 0.078) on stillbirths, suggesting that this haplotype may in fact be associated with stillbirths or in LD with a causative mutation in the region on BTA13:47,952,032–59,026,521. The other high frequency haplotype, hap1244-1 is located on BTA17 within the longer hap419-1, which had a numeric effect stillbirth close to significance (*P* = 0.0519). The extreme deficiency of homozygous individuals for hap1244-1, and the numeric effect of 419–1 indicate a genetic variant affecting stillbirth in the region BTA17: 62,591,773–65,439,957. Other regions that are likely affecting stillbirth are on BTA2, BTA3, BTA6, BTA8, BTA10, BTA18, BTA20, BTA26, BTA27 and BTA28. On BTA2, there are three haplotypes with significant effects, hap136-1 on BTA2:28,668,665–29,822,786, and hap149-14 and hap51-13 in the region BTA2: 48,993,143–54,536,305. Two HHD haplotypes were associated with fertility but not stillbirth, hap106-1 on BTA1 and hap278-418 on BTA10. We did not detect any indication of chromosomal deletions when we looked at log R values. Therefore, it is more likely that the cause is a single mutation or small structural variation not detectable with medium density genotypes.

The 19 haplotypes here identified have frequency ranging from 1.8% to 8.5%. Therefore, their independent effects do not explain a large part of stillbirths in the population, but their cumulative effects are substantial. A lethal mutation with frequency of 5% will under random mating result in a homozygote frequency of 0.05^2^ = 0.0025. If four such mutations are segregating independently in the population, 1% of all inseminations could result in lethality.

The traits studied here are lowly heritable traits and the raw data from the breeding program records are not completely reliable; farmers and AI technicians do the insemination recording routinely and the data has to be filtered extensively before they are used for genetic evaluation. This is especially the case with insemination records. Calving records should be more reliable; the farmer only has to record whether the calf was born alive, died during birth or shortly after birth, or was dead before birth. Errors and the small number of genotyped animals with phenotypes reduces the power of GWAS, which is reflected in no genome-wide significant marker. Despite this, we have found substantial evidence of genomic regions that affect stillbirth and fertility.

Further research should include genotyping of stillborn Icelandic calves. With enough samples, it should be possible to confirm whether these haplotypes are recessive lethals; if stillborn calves are homozygotes and no live homozygotes are observed, there is strong evidence for causative effect. Furthermore, stillborn calves that are homozygotes for these haplotypes, or other carrier animals, could be sequenced. A mutation observed in the region in carrier animals is a strong candidate to be the causal mutation.

The Icelandic Cattle breeding program can immediately use the results of this study in selection of bulls and to assist farmers in mating decisions. However, more research is needed to establish the causative variants behind the effects found in this study. If the causative variant is found, it can be added to the SNP chip used for routine genotyping and the breeding program can publish carrier status of genotyped cows and AI bulls. This would allow farmers to avoid carrier by carrier matings.

## Conclusion

We found 19 HHD haplotypes on 13 autosomes in the Icelandic Cattle populations that had significant effects on stillbirth, or fertility, or both. Two regions, on BTA8 and BTA13, are likely to contain a loss-of-function mutation affecting stillbirth and fertility due to multiple evidence. Further research should include genotypes and, ideally, sequences of stillborn calves and carrier animals.

## Supplementary Information

Below is the link to the electronic supplementary material.

Online Resource 1. Identified segments with homozygous haplotype deficiency. (XLSX 20.6 KB)

Online Resource 2. Effects of HHD haplotypes on stillbirth. (XLSX 16.2 KB)

Online Resource 3. Effect of carrier status on non-return rates at 56 days. (XLSX 29.7 KB)

Online Resource 4. GWAS summary statistics: chromosome, position, effect size, its standard error, and P-value. (XLSX 17.5 KB)

Online Resource 5. Expected and observed distribution of P-values. (DOCX 472 KB)

## Data Availability

The data used for this study is owned by the Farmer’s Association of Iceland. Restrictions apply to the availability of these data, which were used under license for this study. Data is available from the authors with the permission of the Farmer’s Association of Iceland.
