# Supplementary material for: Haplotypes affecting stillbirth and fertility in Icelandic Dairy Cattle
Source: J Appl Genet. 2025 Jun 2;66(4):1053–64. doi: 10.1007/s13353-025-00978-0 (PMC12605402; doi:10.1007/s13353-025-00978-0)

Haplotypes affecting stillbirth and fertility in Icelandic Dairy Cattle. Journal of Applied Genetics. Egill Gautason^1^, Þórdís Þórarinsdóttir^2^ and Goutam Sahana^3^

^1^Agricultural University of Iceland, 311 Borgarnes, Iceland

^2^The Icelandic Agricultural Advisory Center, Óseyri 2, 603 Akureyri, Iceland

^3^Aarhus University, Center for Quantitative Genetics and Genomics, C. F. Møllers Allé 3, 8000 Aarhus, Denmark

[goutam.sahana@qgg.au.dk](mailto:goutam.sahana@qgg.au.dk)

# Daughter stillbirth 1


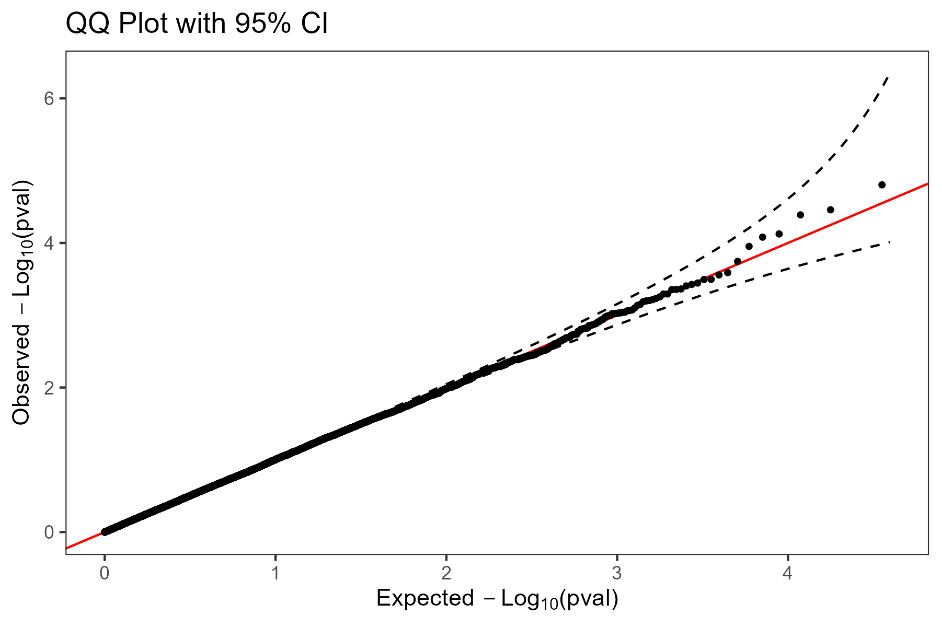


# Sire stillbirth 1


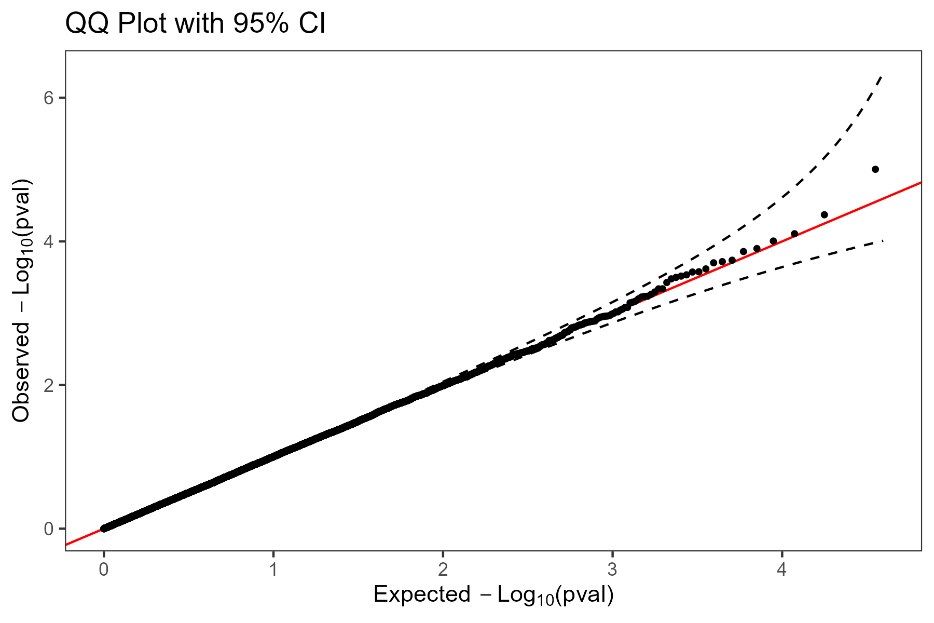


# Daugher stillbirth 2

#
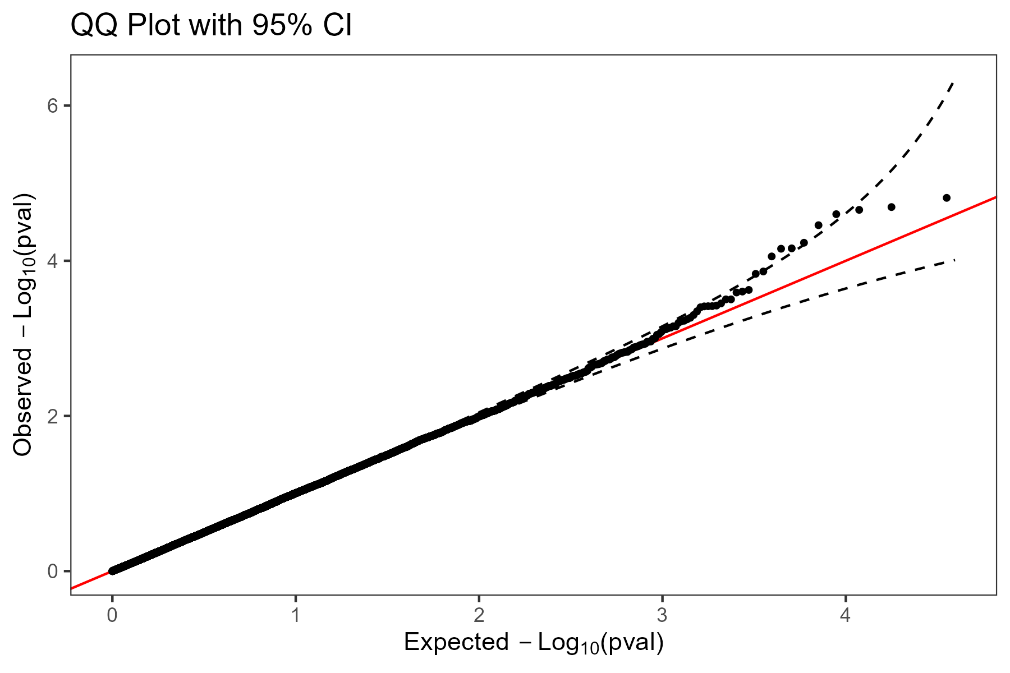


# Sire stillbirth 2

#
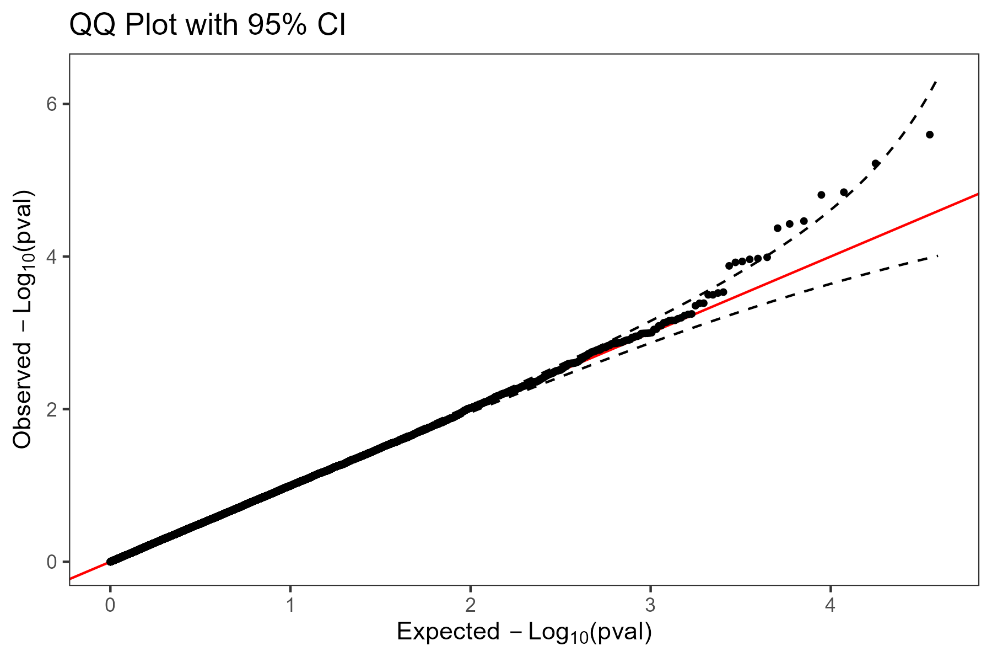


# CI


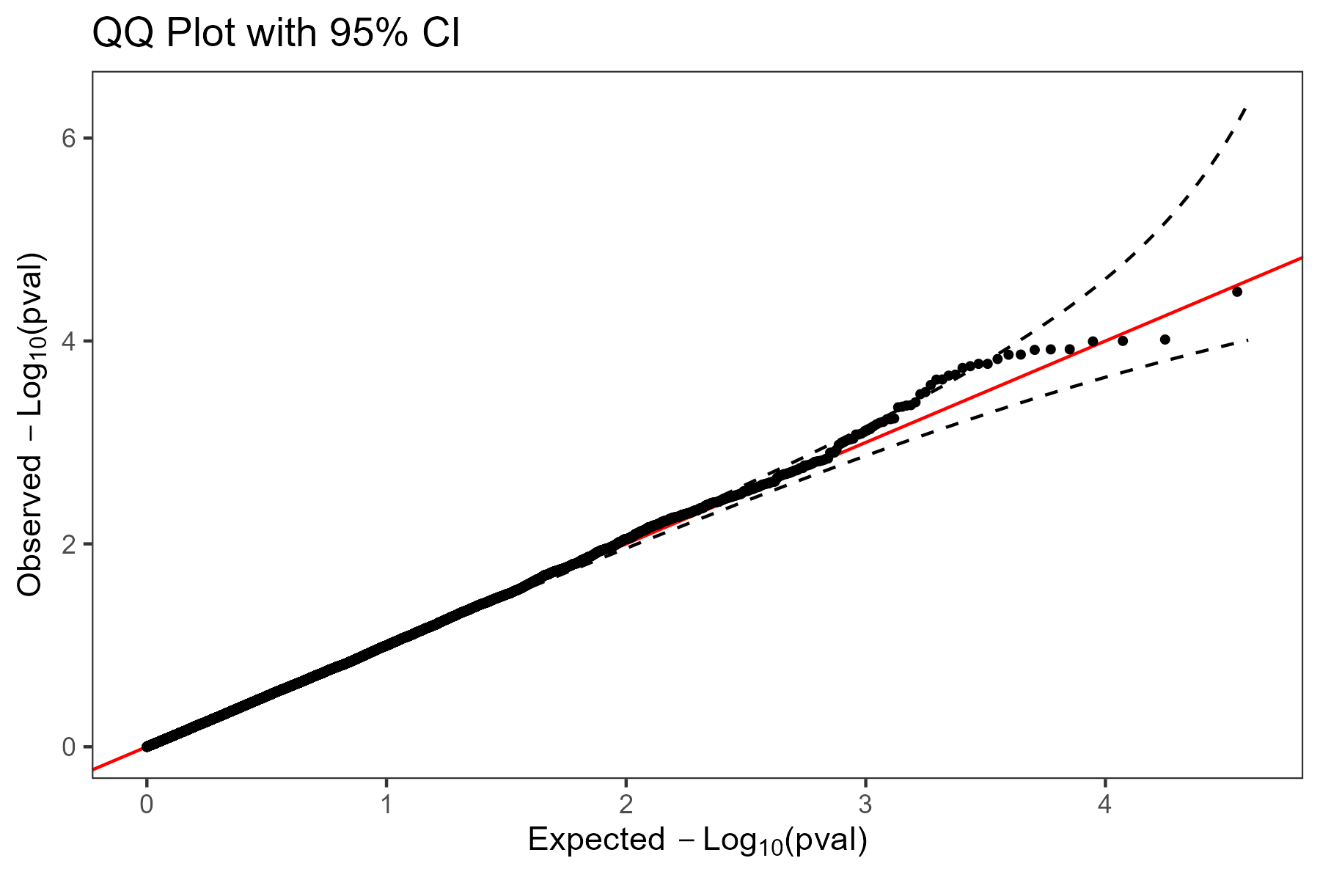
IFL
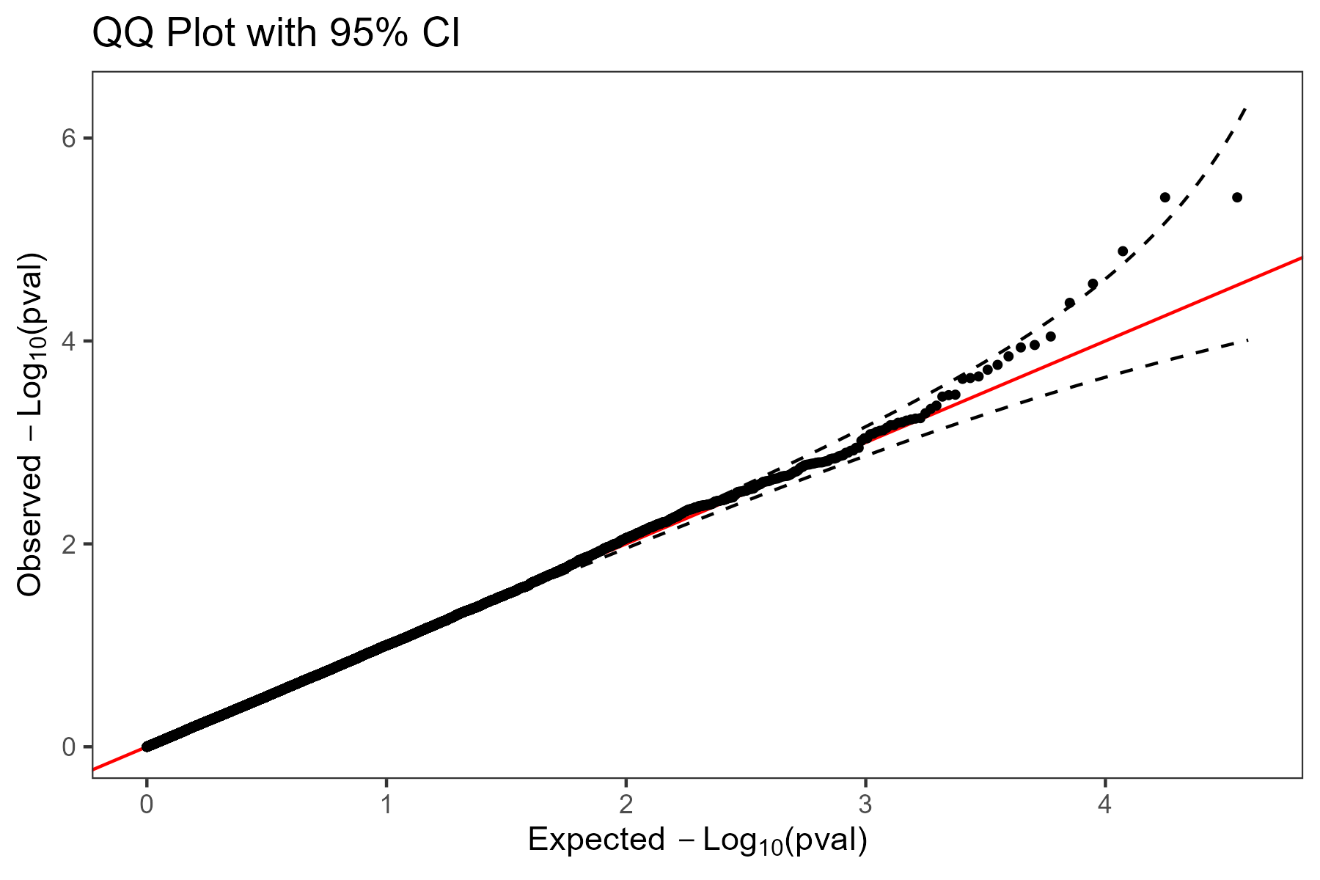


# AIS

#
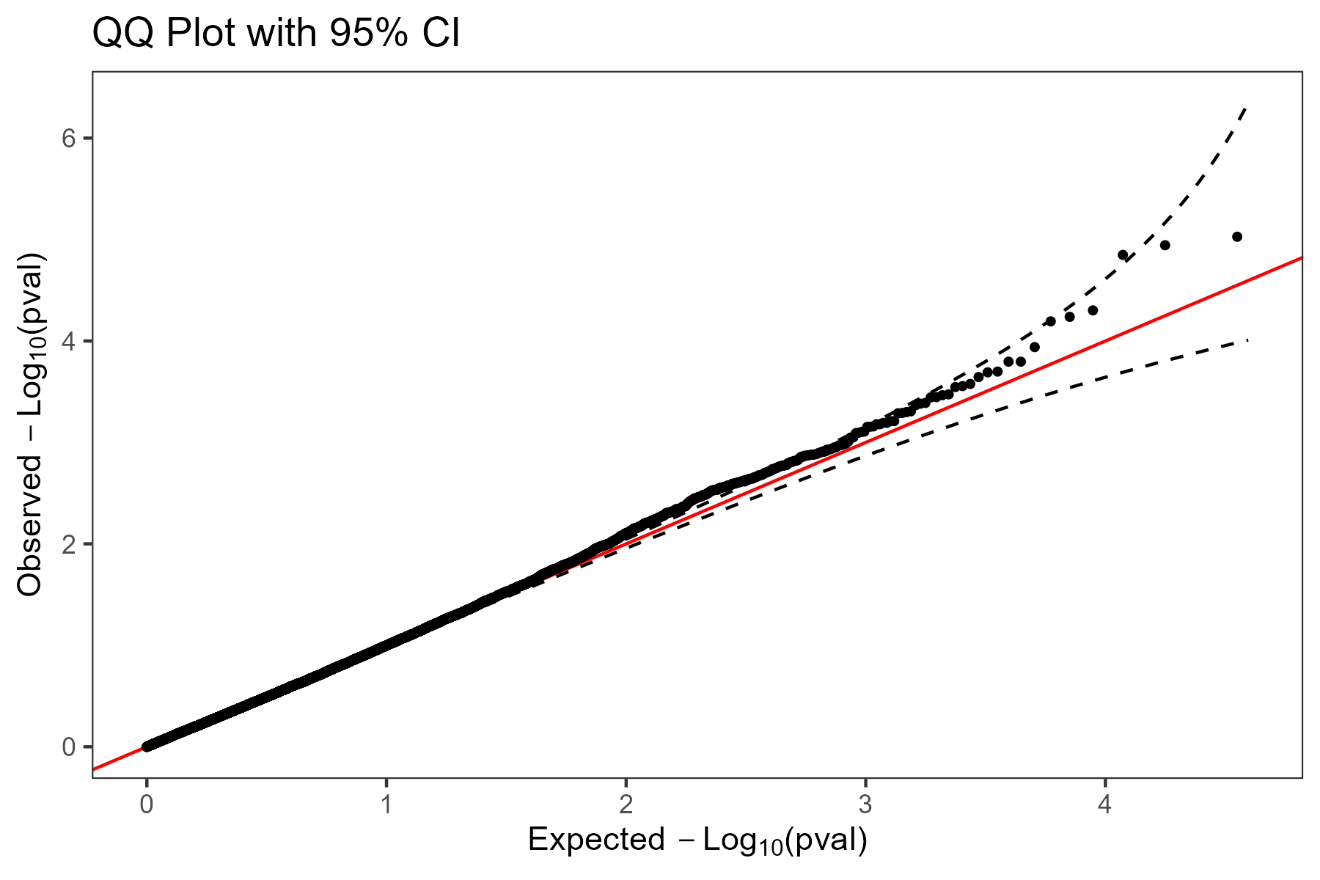


# CR

#
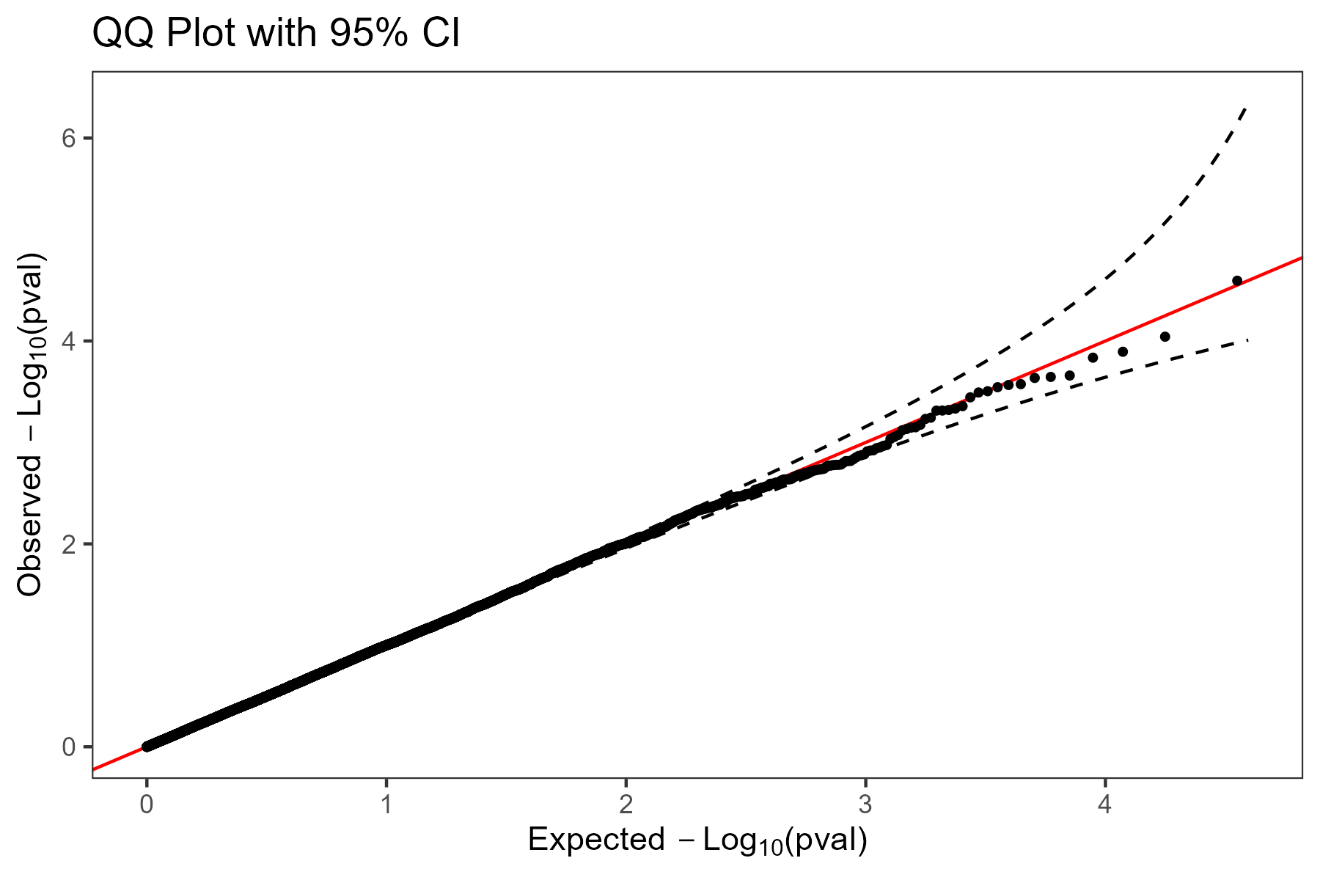


# Infertility


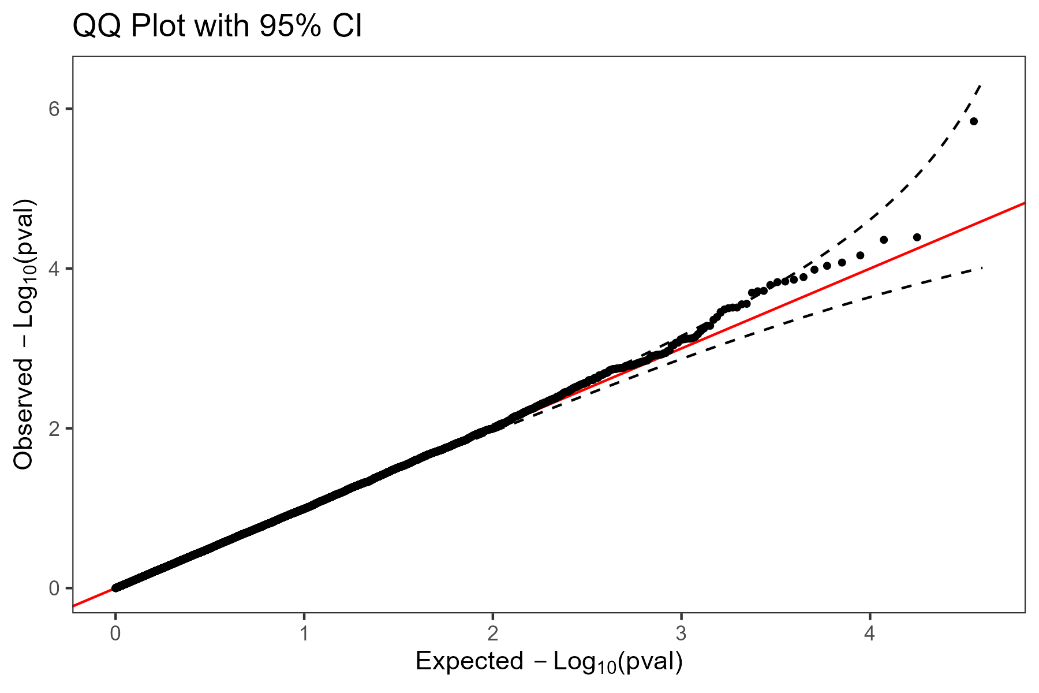

Supplement: Supplementary file 5 — Online Resource 5. Expected and observed distribution of P-values. (DOCX 472 KB) [file 13353_2025_978_MOESM5_ESM.docx]
